# Simultaneous encryption and compression of medical images based on optimized tensor compressed sensing with 3D Lorenz

**DOI:** 10.1186/s12938-016-0239-1

**Published:** 2016-11-04

**Authors:** Qingzhu Wang, Xiaoming Chen, Mengying Wei, Zhuang Miao

**Affiliations:** 1 School of Information Engineering, Northeast Dianli University, Jilin, 132012 China; 2 Department of Neurosurgery, China-Japan Union Hospital of Jilin University, Changchun, China

**Keywords:** Encryption and compression, Hither order singular value decomposition, 3D Lorenz, Medical images

## Abstract

**Background:**

The existing techniques for simultaneous encryption and compression of images refer lossy compression. Their reconstruction performances did not meet the accuracy of medical images because most of them have not been applicable to three-dimensional (3D) medical image volumes intrinsically represented by tensors.

**Methods:**

We propose a tensor-based algorithm using tensor compressive sensing (TCS) to address these issues. Alternating least squares is further used to optimize the TCS with measurement matrices encrypted by discrete 3D Lorenz.

**Results:**

The proposed method preserves the intrinsic structure of tensor-based 3D images and achieves a better balance of compression ratio, decryption accuracy, and security. Furthermore, the characteristic of the tensor product can be used as additional keys to make unauthorized decryption harder.

**Conclusions:**

Numerical simulation results verify the validity and the reliability of this scheme.

## Background

In recent years, numerous studies on encryption of medical images, such as computed tomography (CT) and magnetic resonance imaging (MRI), have been reported [1–6], although most of them did not consider compression during encryption. The storage, transmission, and retrieval of massive bio-information should meet several compulsory requirements [7]: (1) high efficiency for rapid transmission and prompt retrieval; (2) strict information security to guarantee users’ privacy; and (3) high data fidelity to preserve the pathological information. It requires decreasing the quantity of data to be transmitted (compression) and protecting such data against unauthorized access (encryption). Therefore, simultaneous compression and encryption technology of medical images that are represented as three-dimensional (3D) volumes has additional meanings.

Existing simultaneous compression and encryption algorithms are typically applied to ordinal images rather than medical images, because they refer to lossy compression. Alfalou et al. proposed a series of representative algorithms for the simultaneous compression and encryption of 3D images. The latest and most effective algorithm is based on spectral fusion and discrete cosine transform (DCT) [8]. However, the decryption error increases rapidly along with the increase of the number of images, indicating that it cannot handle large amounts of images simultaneously. Emerging algorithms for simultaneous encryption and compression are based mainly on compressed sensing (CS), which can activate compression during the sampling process [9]. An example being Valerio et al., who proposed a multiclass encryption by CS to withstand the common attack [10, 11]. To further enhance security, CS-based encryption algorithms were constructed by combining the chaos map and some optical encryption techniques, such as double random phase encryption (DRPE), fractional Fourier transform (FrFT), and fractional Mellin transform (FrMT) [12–19].

The medical images are different from other images because of their particular properties. There are legal and strict regulations applied to medical multimedia information due to the health of a patient depending on the correctness and accuracy of this information [20]. The quality of the decrypted and compressed data must be adequate to allow for a correct diagnosis when it is reconstructed. However, the existing algorithms did not meet this requirement because most of them have not been applicable to 3D images intrinsically represented by tensors. Conventional CS theory relies on data representation in the form of one-dimensional vectors. Application of CS to higher dimensional data representation is typically performed by conversion of the data to very long vectors that must be measured using very large sampling matrices, thus destroying the intrinsic structure and imposing a huge memory burden.

Recently, Cesar et al. propose a tensor CS (TCS) based on higher order singular value decomposition (HOSVD) that introduces a direct reconstruction formula to recover a tensor from a set of multi-linear projections, which are obtained by multiplying the data tensor by a different sensing matrix in each mode [21]. This HOSVD-based TCS achieved more accurate and efficient reconstruction results when compared to other existing sparsity-based TCS methods [22–24]. Indeed, we believe that, if it was used to design TCS-based encryption of 3D images better performance would occur.

We further introduce alternating least squares (ALS) into HOSVD-based TCS [21], and control the measurement matrices by 3D Lorenz [25–27]. Such a simultaneous compression and encryption algorithm for 3D medical images has two main advantages: (1) the preservation of intrinsic structures of the tensor data for the purpose of reducing the decryption error and increasing the compression ratio; (2) the keys consist of those generated by tensor decomposition, and 3D Lorenz. Particularly, the order of the tensor product used in the TCS can be used as additional keys to make unauthorized decryption harder.

This paper is organized as follows: in “Theory” section, the related notation, definition and basic results used throughout the paper, are introduced; in “Proposed encryption” section, the encryption and decryption algorithms are proposed; in “Numerical simulation results” section, several numerical results based on 3D lung CT images are provided, to corroborate our theoretical results and evaluate the stability and robustness of our proposed scheme, in “Conclusion” section, the main conclusions drawn from the present work are outlined.

## Theory

### HOSVD

The higher-order singular value decomposition (HOSVD) provides a generalization of the low-rank approximation of matrices to the case of tensors [28–30]. To facilitate the distinction between scalars, vectors, matrices and higher dimensional tensors, the type of a given quantity will be reduced by its representation: scalars are denoted by lower-case letters ($a$), vectors are written as capitals (${\mathbf{a}}$), matrices corresponding to bold-face capitals (${\mathbf{A}}$) and tensors are written as calligraphic letters (${\mathbf{\mathcal{A}}}$).

A tensor is a multidimensional array with the number of modes represented by the tensor order. For instance, tensor ${\mathbf{\mathcal{A}}} \in R^{{M_{1} \times \cdots \times M_{d} }}$ has order $d$ and the dimension of its *i*-th mode is $M_{i}$.

1. Mode-$i$ product: A mode-$i$ product of a tensor ${\mathbf{\mathcal{A}}}$ and a matrix $\varPhi \in R^{{M_{i} \times m}}$ is denoted by ${\mathbf{\mathcal{A}}} \times_{i} \varPhi$ and is of size $m \times \left( {M_{1} \cdots M_{i - 1} \cdot M_{i + 1} \cdots M_{d} } \right)$ matrix.
2. Mode-$i$ unfolding: The mode-$i$ unfolding ${\mathbf{A}}_{(i)}$ of ${\mathbf{\mathcal{A}}}$ arranges the mode-$i$ fibers to be the columns of the resulting matrix.
3. HOSVD: The decomposition and reconstruction of ${\mathbf{\mathcal{A}}}$ can be written as the product:
  1 $$\left\{ \begin{aligned} {\mathbf{\mathcal{W}}} &= {\mathbf{\mathcal{A}}} \times_{1} \varPhi_{1}^{T} \cdots \times_{{M_{d} }} \varPhi_{{M_{d} }}^{T} \\ {\mathbf{\overset{\lower0.5em\hbox{$\smash{\scriptscriptstyle\frown}$}}{\mathcal{A}} }} &= {\mathbf{\overset{\lower0.5em\hbox{$\smash{\scriptscriptstyle\frown}$}}{\mathcal{W}} }} \times_{1} \varPhi_{1} \cdots \times_{{M_{d} }} \varPhi_{{M_{d} }} \\ \end{aligned} \right.$$
  where $\varPhi_{i} \in R^{{M_{i} \times m_{i} }}$, and ${\mathbf{\mathcal{W}}}$ is a complex $\left( {m_{1} \times m_{2} \times \cdots \times m_{d} } \right)$-tensor of which the subtensors obtained by corresponding singular values.
4. Tucker-TCS: in [21], a more stable, robust and accuracy tensor reconstruction of CS is proposed:
  2 $${\mathbf{\overset{\lower0.5em\hbox{$\smash{\scriptscriptstyle\frown}$}}{\mathcal{A}} }}\text{ = }{\mathbf{\overset{\lower0.5em\hbox{$\smash{\scriptscriptstyle\frown}$}}{\mathcal{W}} }} \times_{1} {\mathbf{Z}}_{1} {\mathbf{W}}_{(1)}^{\dag } \cdots \times_{{M_{d} }} {\mathbf{Z}}_{{M_{d} }} {\mathbf{W}}_{{(M_{d} )}}^{\dag }$$
  where “$\dag$” stands for the MP pseudo-inverse of a matrix. We assume that the following sets of compressive multi-way measurements ${\mathbf{Z}}^{(n)}$ are available:
  3 $${\mathbf{Z}}^{(n)} = {\mathbf{\mathcal{A}}} \times_{1} \varPhi_{1}^{T} \times_{2} \cdots \times_{n - 1} \varPhi_{n - 1}^{T} \times_{n + 1} \varPhi_{n + 1}^{T} \times_{n + 2} \cdots \times_{{M_{d} }} \varPhi_{{M_{d} }}^{T}$$
  4 $${\mathbf{Z}}_{n} = \left( {{\mathbf{Z}}^{(n)} } \right)_{(n)}$$

### 3D Lorenz

Mohmad et al. [25, 26] propose a 3D discrete Lorenz system, which has a high order and also low complexity when implemented in digital hardware. The discrete Lorenz attractor employed here is given by the following difference equations

5 $$\left\{ \begin{aligned} U_{k + 1} &= g1\left( {A\left( {V_{k} - U_{k} } \right)} \right) + U_{k} \hfill \\ V_{k + 1} &= g2(BU_{k} - V_{k} - 20U_{k} W_{k} ) + V_{k} \hfill \\ W_{k + 1} &= g3(5U_{k} V_{k} - CW_{k} ) + W_{k} \hfill \\ \end{aligned} \right.$$

where *U, V, W* are three state variables, *A, B, C* are parameters, and $g1,\;g2,\;g3$ are gains (step size). The calculating method of (5) is finite difference.

## Proposed encryption

**Fig. 1:**
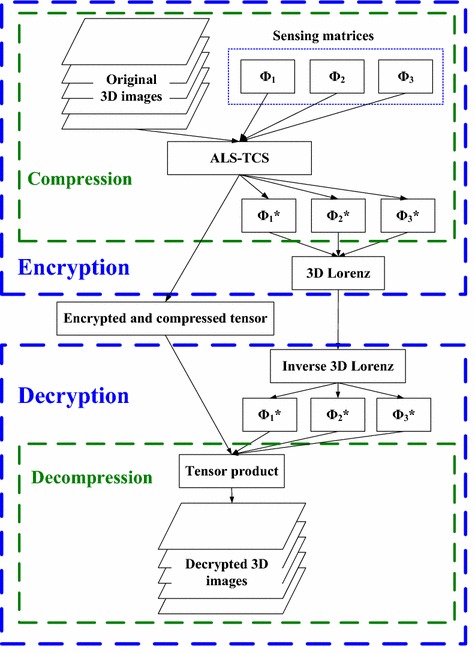
Illustration of the proposed simultaneous compression and encryption system of 3D images

The proposed system can be split into encryption and decryption algorithms as illustrated in Fig. 1. The compression and decompression procedures are embedded in the encryption and decryption, respectively.

### Encryption

For the initial 3D image ${\mathbf{\mathcal{A}}} \in R^{{M_{1} \times M_{2} \times M_{3} }}$, the encryption process consists of the following steps:

1. Initialize randomly the three Gaussian sensing matrices $\varPhi_{i}^{(0)} \in R^{{M_{i} \times m_{i} }}$ $(m_{i} < M_{i} ,\;i = 1,2,3)$. To accurately decrypt ${\mathbf{\mathcal{A}}}$, the optimal $\varPhi_{i}^{{}}$ should satisfy:
  6 $$\{ \varPhi_{1}^{*} ,\;\varPhi_{2}^{*} ,\;\varPhi_{3}^{*} \} = \arg \mathop {\hbox{min} }\limits_{{}} \left\| {{\mathbf{\mathcal{A} }} - {\mathbf{\mathcal{W}}} \times_{1} \varPhi_{1} \times_{2} \varPhi_{2} \times_{3} \varPhi_{3} } \right\|_{F}^{2}$$
  As in [28], this problem can be converted to
  7 $$\{ \varPhi_{1}^{*} ,\;\varPhi_{2}^{*} ,\;\varPhi_{3}^{*} \} = \arg \mathop {\hbox{max} }\limits_{{}} \left\| {{\mathbf{\mathcal{A}}} \times_{1} \varPhi_{1}^{T} \times_{2} \varPhi_{2}^{T} \times_{3} \varPhi_{3}^{T} } \right\|_{F}^{2}$$
  To solve this problem, it is sufficient to find $\varPhi_{i}$’s satisfying $\varPhi_{i}^{T} \varPhi_{i} = I$. The reconstruction algorithm as Eq. (2) of HOSVD-based TCS achieved a more accurate solution than that as Eq. (1) of HOSVD. To further improve the reconstruction accuracy, we use an alternating least squares (ALS) approach to solve Eq. (7).
2. For $k = 0$, iterate Eqs. (8)–(11) until $\varPhi_{i}^{{}}$ converges or the maximum iteration is achieved:
  8 $$\left\{ \begin{aligned} {\mathbf{Z}}^{(1)} = {\mathbf{\mathcal{A}}} \times_{2} \varPhi_{2}^{(k)T} \times_{3} \varPhi_{3}^{(k)T} \hfill \\ {\mathbf{Z}}^{(2)} = {\mathbf{\mathcal{A}}} \times_{1} \varPhi_{1}^{(k)T} \times_{3} \varPhi_{3}^{(k)T} \hfill \\ {\mathbf{Z}}^{(3)} = {\mathbf{\mathcal{A}}} \times_{1} \varPhi_{1}^{(k)T} \times_{2} \varPhi_{2}^{(k)T} \hfill \\ \end{aligned} \right.$$
  9 $${\mathbf{Z}}_{i} = \left( {{\mathbf{Z}}^{(i)} } \right)_{(i)}$$
  10 $${\mathbf{Z}}_{i} \approx {\mathbf{U}}_{{i,m_{i} }} {\varvec{\Sigma}}_{{i,m_{i} }} {\mathbf{V}}_{{i,m_{i} }}^{T}$$
  where ${\varvec{\Sigma}}_{{m_{i} }} = diag\left\{ {\sigma_{1} , \ldots ,\sigma_{{m_{i} }} } \right\}$ is the diagonal matrix containing the $m_{i}$ largest singular value $\sigma_{1} \ge \sigma_{2} \ge \cdots \ge \sigma_{{m_{i} }}$ of ${\mathbf{Z}}_{i}$, and ${\mathbf{U}}_{{m_{i} }}$ and ${\mathbf{V}}_{{m_{i} }}^{{}}$ are matrices whose columns are the leading $m_{i}$ left and right singular vectors of ${\mathbf{Z}}_{i}$, respectively. Let
  11 $$\varPhi_{i}^{(k + 1)} = {\mathbf{U}}_{{i,m_{i} }}$$
  Then the optimal $\varPhi_{1}^{*}$, $\varPhi_{2}^{*}$ and $\varPhi_{3}^{*}$ are obtained.
3. Compute the compressed core tensor
  12 $${\mathbf{\mathcal{W}}} = {\mathbf{\mathcal{A}}} \times_{1} \varPhi_{1}^{T} \times_{2} \varPhi_{2}^{T} \times_{3} \varPhi_{3}^{T}$$
4. Unfold ${\mathbf{\mathcal{W}}}$ into its *n*-mode ${\mathbf{W}}_{(n)}$. The mode *n* is a private key which has three possible values: 1, 2 and 3.
5. $\varPhi_{1}$, $\varPhi_{2}$ and $\varPhi_{3}$ are synchronously constructed by 3D Lorenz as Eq. (5).
  13 $$E_{{\varPhi_{i} }} = L\left( {\varPhi_{i} } \right)$$
  where $E_{{\varPhi_{i} }} \in R^{{M_{i} \times m_{i} }}$. Hence the compression ratio is given by:
  14 $$\gamma = \frac{{m_{1} m_{2} m_{3} + m_{1} M_{1} + m_{2} M_{2} + m_{3} M_{3} }}{{M_{1} M_{2} M_{3} }} \approx \frac{{m_{1} m_{2} m_{3} }}{{M_{1} M_{2} M_{3} }}$$

The details of how to synchronize the image by 3D Lorenz system are introduced below. One data sample $\varphi_{i,k}$ is inserted into *U*, which gives

15 $$\left\{ \begin{aligned} U_{k + 1} &= g1\left( {A\left( {V_{k} - U_{k} } \right) + \varphi_{i,k} } \right) + U_{k} \hfill \\ V_{k + 1} &= g2(BU_{k} - V_{k} - 20U_{k} W_{k} ) + V_{k} \hfill \\ W_{k + 1} &= g3(5U_{k} V_{k} - CW_{k} ) + W_{k} \hfill \\ \end{aligned} \right.$$

where $\varphi_{i,k}$ is the *k*-th element of $\phi_{i}$($\phi_{i}$ is the vectorization of $\varPhi_{i}$, i.e. $\phi_{i} = vec\left( {\varPhi_{i} } \right)$). The initial conditions $U_{0} ,\;V_{0} ,\;W_{0} ,\;$ parameters *A*, *B*, *C*, and *g*1, *g*2, *g*3 are known by both transmitter and receiver. The transmitted signal is the *U* state variable, and the objective is to retrieve $\varphi_{i,k}$ from this signal at the receiver. Feedback is used to update the state variables at the receiver to synchronize the system and allow decryption of subsequent data values.

16 $$\tilde{\varphi }_{i,k} = \frac{{U_{k + 1} - U_{k} }}{g1} - A(V_{k} - U_{k} )$$

After $\tilde{\varphi }_{i,k}$ is obtained, the receiver state equations can be updated, thus achieving synchronization with the transmitter.

17 $$\left\{ \begin{aligned} U_{k + 1} &= g1\left( {A\left( {V_{k} - U_{k} } \right) + \tilde{\varphi }_{i,k} } \right) + U_{k} \hfill \\ \tilde{V}_{k + 1} &= g2(BU_{k} - V_{k} - 20U_{k} W_{k} ) + V_{k} \hfill \\ \tilde{W}_{k + 1} &= g3(5U_{k} V_{k} - CW_{k} ) + W_{k} \hfill \\ \end{aligned} \right.$$

### Decryption

The decryption process consists of the following steps:

1. $E_{{\varPhi_{i} }}$ are inverse transformed by 3D Lorenz:
  18 $$D_{{\varPhi_{i} }} = L^{ - 1} \left( {E_{{\varPhi_{i} }} } \right)$$
$L^{ - 1} \left( \cdot \right)$ is computed during Eq. (16).
2. ${\mathbf{W}}_{(n)}$ and the obtained $D_{{\varPhi_{i} }}$ are multiplied in the correct order to recover ${\mathbf{\mathcal{A}^{\prime}}}$. There are three feasible ways to achieve this:
  19 $${\mathbf{A^{\prime}}}_{(n)} = \left\{ \begin{aligned} D_{{\varPhi_{1} }} \cdot {\mathbf{W}}_{(1)} \cdot \left( {D_{{\varPhi_{2} }} \otimes D_{{\varPhi_{3} }} } \right)^{T} \hfill \\ D_{{\varPhi_{2} }} \cdot {\mathbf{W}}_{(2)} \cdot \left( {D_{{\varPhi_{3} }} \otimes D_{{\varPhi_{1} }} } \right)^{T} \hfill \\ D_{{\varPhi_{3} }} \cdot {\mathbf{W}}_{(3)} \cdot \left( {D_{{\varPhi_{1} }} \otimes D_{{\varPhi_{2} }} } \right)^{T} \hfill \\ \end{aligned} \right.$$
  where ‘$\otimes$’ represents the Kronecker product.
3. Then, fold ${\mathbf{A^{\prime}}}_{(n)}$ into ${\mathbf{\mathcal{A}^{\prime}}}$ according to the private key *n.*

It is obvious that besides the secret keys of measurement matrices, the unfolding model *n* (order of tensor product) can be used as an additional key.

## Numerical simulation results

Numerical simulations were conducted with Matlab2011 on a work station with an Intel Core i7 CPU and 64 GB RAM. The decryption error and compression ratio of the proposed system are introduced in “Decryption accuracy and compression ratio” section, the histograms are analyzed in “Histograms and statistical analysis” section, the secret keys are illustrated in “Rate-distortion” section, and the robustness is stated in “Secret keys” section.

### Decryption accuracy and compression ratio

Our experiments are conducted on lung CT sequences in lung image database consortium (LIDC) [31]. Each frame of one CT sequence is preprocessed to have 512 × 512, where 512 frames were chosen. The CT sequence together is represented by a 512 × 512 × 512 tensor and has 134,217,728 voxels in total. The randomly constructed Gaussian measurement matrix for each mode is now of size $512 \times m_{i}$ ($i = 1,2,3$). Thus, the compression ratio is given by:

$$\gamma = \frac{{m_{1} m_{2} m_{3} }}{512 \times 512 \times 512}$$

The quantitative measure of the decryption error is the peak signal noise ratio (PSNR), which is based on the root mean square error (RMSE) between the decrypted data and ground truth and can be represented as:

20 $$PSNR = 20\log_{10} \frac{255}{RMSE}$$

To further evaluate the performance of decryption, the structural similarity index (SSIM) is used as another indicator.

In this section, we compare the proposed algorithm with state of the art algorithms to show its superiority. These algorithms are presented briefly as follows:

1. As demonstrated previously [13], an encryption based on 2D_CS in the FrMT domain (algorithm 1) stands out for its efficient, robust, and secure encryption performance. In this algorithm, the 2D CS is based on a 2D wavelet, measuring matrices with Logistic map and a 2D NSL_0_ reconstruction algorithm. Notably, although the security of FrMT is better than that of FrFT, its decryption accuracy is less than the later. In order to verity the decryption accuracy and compression ratio of our tensor-based algorithm, we replaced FrMT with FrFT in algorithm 1.
2. Additionally, we chose an encryption algorithm based on HOSVD-TCS [21] with FrFT (algorithm 2).
3. Also, as previously shown [8], an encryption algorithm based on spectral fusion and DCT obtained a better PSNR when compared with previous compression-encryption implementations. Accordingly, this became algorithm 3.

**Fig. 2:**
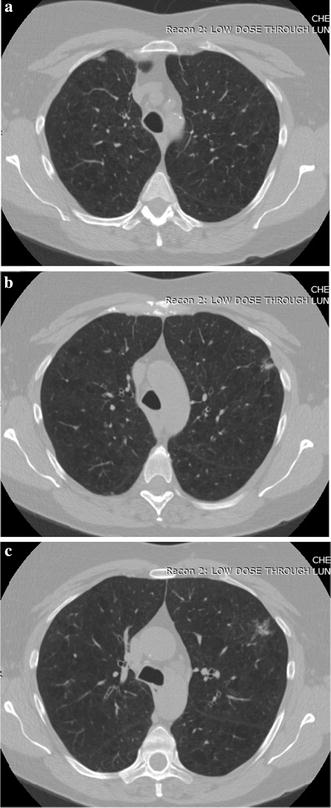
Original CT frames.** a** Frame 117,** b** Frame 138,** c** Frame 159

**Fig. 3:**
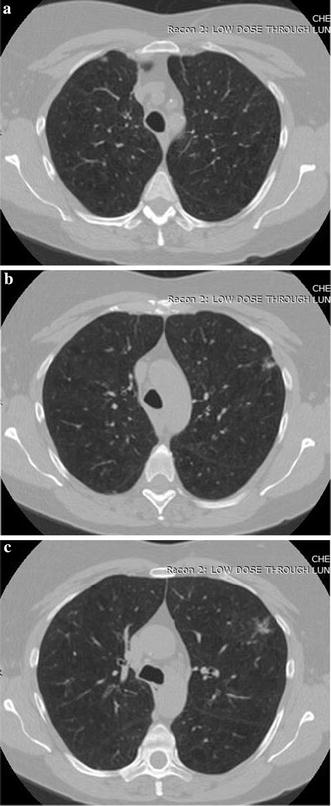
Decrypted frames by the proposed algorithm.** a** Frame 117,** b** Frame 138,** c** Frame 159

**Fig. 4:**
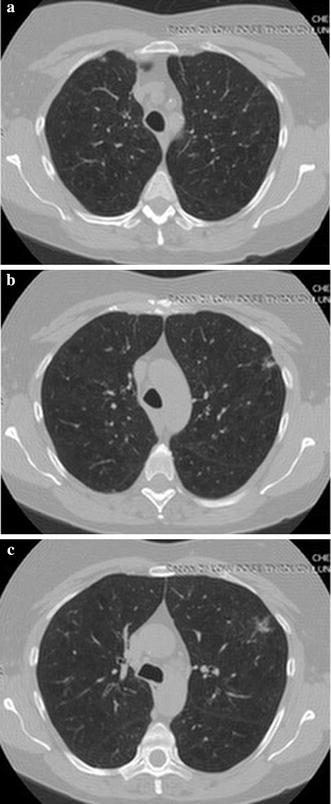
Decrypted frames by algorithm 3.** a** Frame 117,** b** Frame 138,** c** Frame 159

**Fig. 5:**
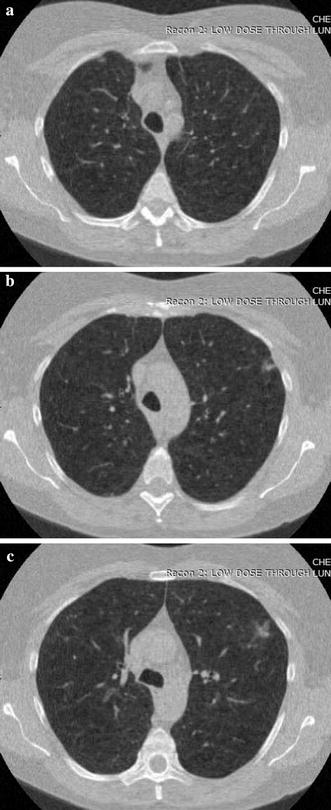
Decrypted frames by algorithm 2.** a** Frame 117,** b** Frame 138,** c** Frame 159

**Fig. 6:**
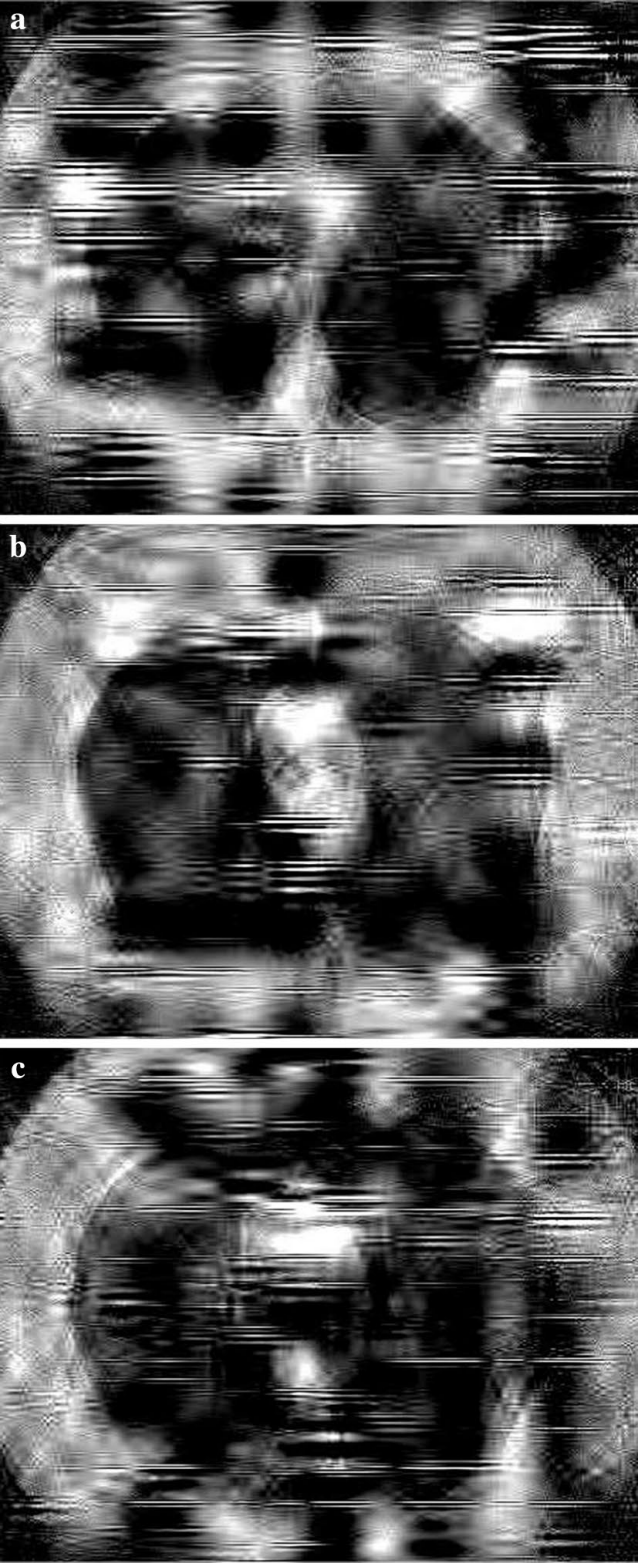
Decrypted frames by algorithm 1.** a** Frame 117,** b** Frame 138,** c** Frame 159

**Fig. 7:**
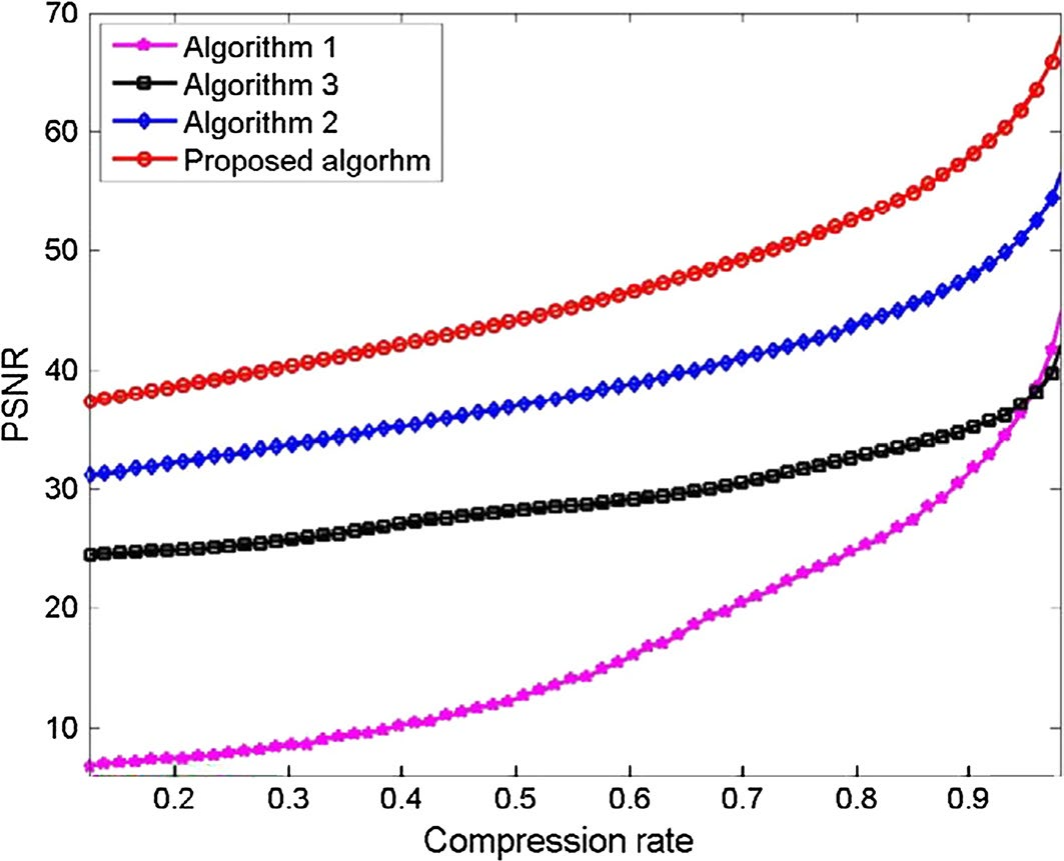
PSNR (dB) comparison of the four algorithms at compression ratio values ranging from 0.125 to 0.98

A visual evaluation of the decryption results under frames 117, 138 and 159 of one CT sequence at the compression $\gamma = 0.125$ is shown in Figs. 2, 3, 4, 5, 6. As shown in Fig. 3, all the tissues within the lung volume are clear. Our clinical experts did not find distinct differences between the decrypted and the original CT images. The quantitative summaries of the above algorithms are shown in Tables 1, 2 and Fig. 7, where the advantages of the proposed algorithm are highlighted. It is evident that the advantages of the proposed algorithm over the other methods increase with the compression ratio. Case in point is in algorithm 3, where improving the compression ratio requires a large number of frames. However, the PNSR rapidly decreases with the increase of the number of frames. Thus, the algorithm cannot handle large number of frames.

**Table 1:** PSNR at different compression ratio of the CT sequence

Methods	Compression ratio					
	$\frac{{(256)^{3} }}{{(512)^{3} }} = 0.125$	$\frac{{(352)^{3} }}{{(512)^{3} }} = 0.33$	$\frac{{(384)^{3} }}{{(128)^{3} }} = 0.42$	$\frac{{(416)^{3} }}{{(512)^{3} }} = 0.54$	$\frac{{(448)^{3} }}{{(512)^{3} }} = 0.67$	$\frac{{(480)^{3} }}{{(512)^{3} }} = 0.82$
Algorithm 1	6.72	11.26	12.22	19.29	24.00	30.49
Algorithm 2	31.12	36.12	38.00	40.34	43.01	47.28
Algorithm 3	24.55	27.78	28.74	30.03	32.30	34.67
Proposed algorithm	37.76	43.17	45.56	48.43	51.95	57.26

**Table 2:** Comparison of SSIM between the proposed algorithm and state to the art methods

Methods	Compression ratio					
	$\frac{{(256)^{3} }}{{(512)^{3} }} = 0.125$	$\frac{{(352)^{3} }}{{(512)^{3} }} = 0.33$	$\frac{{(384)^{3} }}{{(128)^{3} }} = 0.42$	$\frac{{(416)^{3} }}{{(512)^{3} }} = 0.54$	$\frac{{(448)^{3} }}{{(512)^{3} }} = 0.67$	$\frac{{(480)^{3} }}{{(512)^{3} }} = 0.82$
Algorithm 1	0.1406	0.2198	0.3060	0.4378	0.6090	0.8000
Algorithm 2	0.8293	0.9004	0.9195	0.9157	0.9259	0.9312
Algorithm 3	0.7474	0.8664	0.8945	0.9165	0.9314	0.9474
Proposed algorithm	0.9122	0.9468	0.9508	0.9533	0.9544	0.9549

We also compared the computation times (the average computation time of the experiments with all compression ratio and noise level) required in each case. The comparison shows that algorithm 3 provides a much faster computation (Table 3), while the proposed algorithm requires slightly longer computation time because it contains a procedure of iteration. Fortunately, this iteration is much simpler than those of algorithms 1 and 2.

**Table 3:** Comparison of computation time (s) between the proposed algorithm and state to the art methods

Methods	Computation time (s)
Algorithm 1	896
Algorithm 2	188
Algorithm 3	164
Proposed algorithm	220

### Histograms and statistical analysis

#### Histograms

**Fig. 8:**
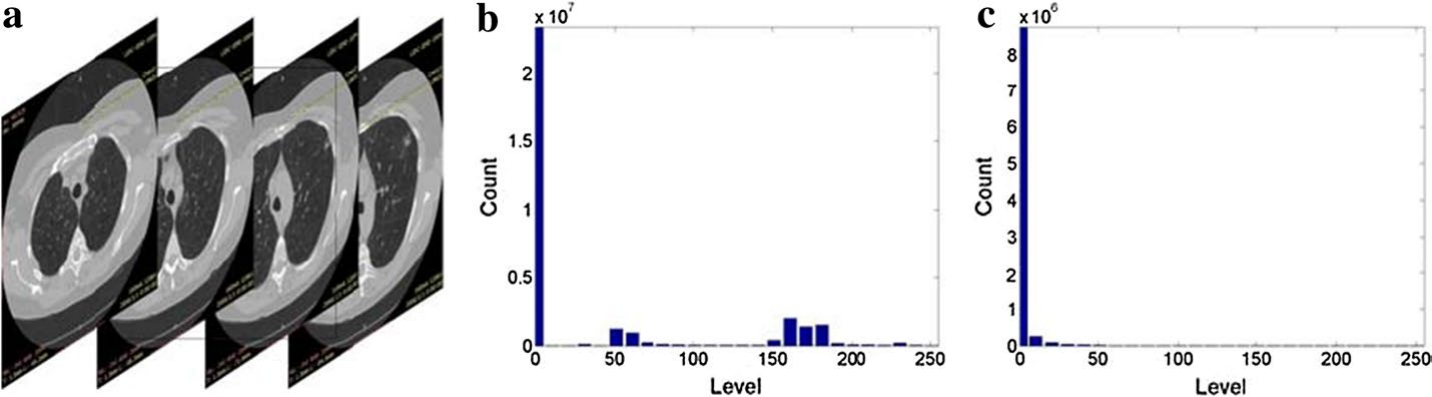
Histograms of 3D images and encrypted parts of CT No. 1.** a** Original CT sequence No.1.** b** Histogram of original CT.** c** Histogram of ciphertext

**Fig. 9:**
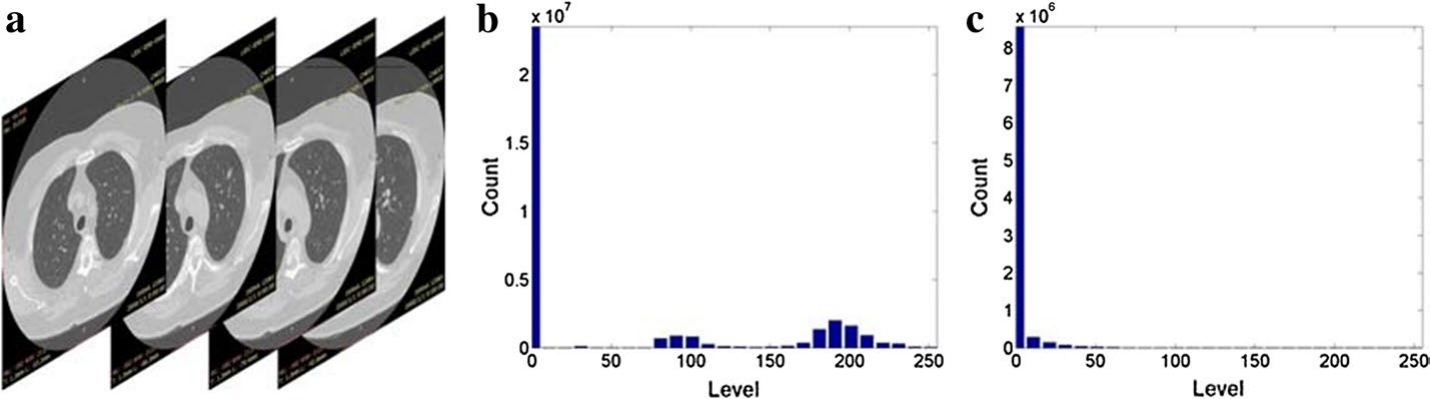
Histograms of 3D images and encrypted parts of CT No. 2.** a** Original CT sequence No.1.** b** Histogram of original CT.** c** Histogram of ciphertext

The histograms of two CT sequences and the encrypted images of their composed parts are shown in Figs. 8 and 9, respectively. The intensity distribution of the histograms of the encrypted images is completely dissimilar from that of the histogram of the original CT, which indicates that an intruder cannot perceive any useful information based on statistical properties. The histograms of the two original CT sequences are evidently different from each other, whereas the histograms of their corresponding encrypted images are similar. The security analysis effectively illustrates the robustness of the proposed method.

#### Statistical analysis

Statistical properties of the images can also be evaluated by the computation of the correlation between two adjacent pixels. By selecting randomly *P* pixels of the image, the correlation coefficient is computed as:

21 $$r_{xy} = \frac{{\text{cov} (x,y)}}{{\sqrt {D(x)D(y)} }}$$

where $x$ is the value of a selected pixel and $y$ is the value of the correspondent adjacent pixel, $D(x)$ is the mean square error.

It is expected that an image will have a correlation coefficient close to 1 before being submitted to the encryption; it is desirable that the correlation coefficient of a ciphered image be as close to 0 as possible. In Table 4, where the simulation results for *P* = 125,000 are shown, we verify that the above described premise is satisfied. This indicates that the proposed encryption scheme is secure against statistical attacks.

**Table 4:** Correlation coefficients of the original volume ($r_{xy}$) and the corresponding ciphered volume ($\tilde{r}_{xy}$); (v), (h) and (d) are related to vertical, horizontal and diagonal adjacency respectively

$r_{xy} (v)$	$\tilde{r}_{xy} (v)$	$r_{xy} (h)$	$\tilde{r}_{xy} (h)$	$r_{xy} (d)$	$\tilde{r}_{xy} (d)$
0.9838	−0.0006	0.9863	−0.0002	0.9958	0.0005

#### Normalized entropy

The normalized entropy [6] of the ciphered image is defined as

22 $$\bar{H} = \frac{{\sum\nolimits_{i = 0}^{P - 1} {\frac{{N_{i} }}{N}\log \frac{N}{{N_{i} }}} }}{{\log_{2} P}}$$

where *P* is the number of different values that the pixels of the ciphered image can assume, $N_{i}$ is the amount of pixels of the ciphered image that assume value *i*, and N is the total amount of pixels of the ciphered image. During the experiments, we found that the ciphered pixels’ intensity of all the algorithms mentioned in the paper are almost equiprobable, so their normalized entropy are all approximate to 1.

### Rate-distortion

Rate-distortion (RD) is an important indicator to evaluate the performance of compression. In a previous study [32], the following mathematical model of CS RD was constructed:

23 $$D^{CS} \left( R \right) = \gamma \frac{\mu }{\mu - 1}\sigma_{\theta }^{2} 2^{ - 2R}$$

**Fig. 10:**
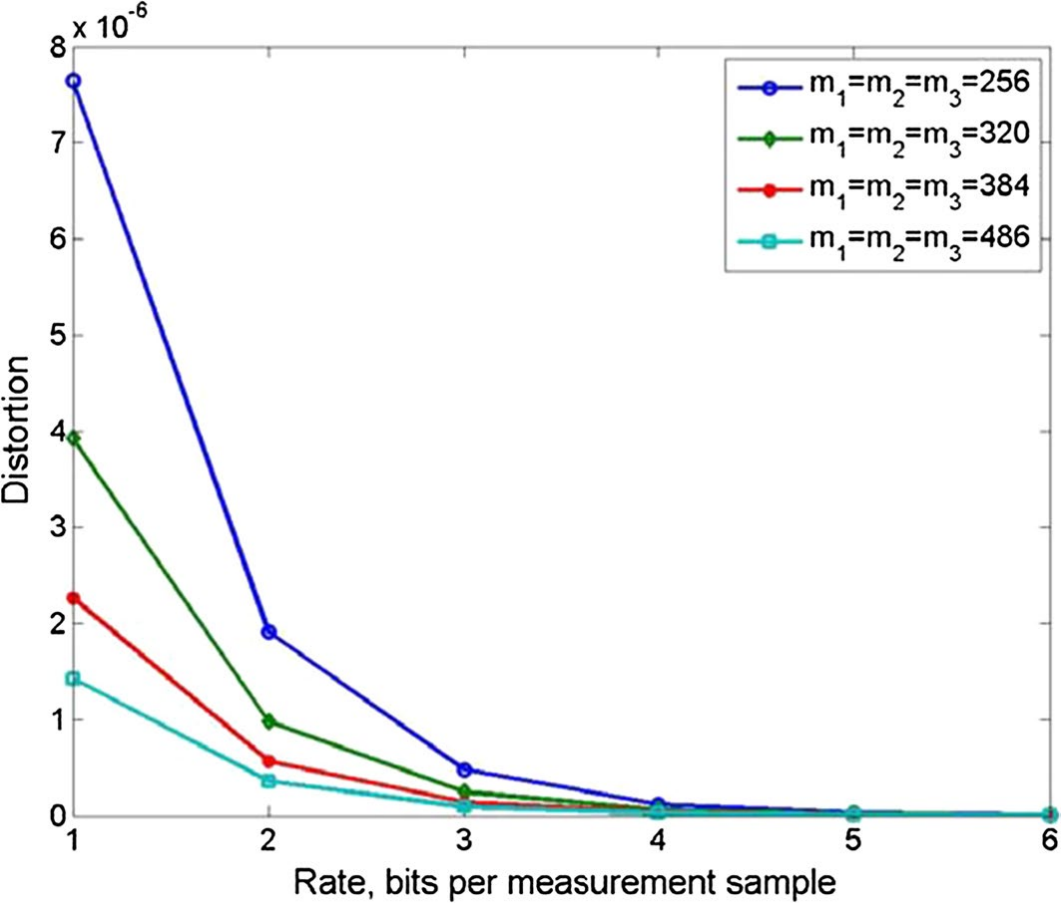
RD diagram at varying compression ration

The proposed TCS-based algorithm falls into the CS category, so the model in Eq. (23) applies for our algorithm. Because there are some differences between traditional vector-based CS and the proposed tensor-based CS, we recalculate some parameters as follows:

24 $$D^{TCS} \left( R \right) = \gamma^{\prime}\frac{{\mu^{\prime}}}{{\mu^{\prime} - 1}}\sigma_{\varPhi }^{2} 2^{ - 2R}$$

where $\gamma^{\prime} = \frac{K}{{m_{1} m_{2} m_{3} }}$, $\mu^{\prime} = \frac{{M_{1} M{}_{2}M_{3} }}{K}$, $\sigma_{\varPhi }^{{}}$ is the singular value of the measurement matrix and $R$ is the rate. It is important to note that in 3D case, there is $\sigma_{{\varPhi_{1} }}^{{}} \approx \sigma_{{\varPhi_{2} }}^{{}} \approx \sigma_{{\varPhi_{3} }}^{{}}$ because the three matrices obey a uniform Gaussian distribution. The RD diagram at several compression ratio points is shown in Fig. 10.

### Secret keys

#### Key space

The keys of the proposed algorithm consist of those generated by the three measurement matrices with 3D Lorenz and the unfolding mode $n$ of the core tensor ${\mathbf{\mathcal{W}}}$. The Key that are used on $\varPhi_{i}$ with 3D Lorenz are the initial values for the three state variables, the $U_{0} ,\;V_{0}$ and $W_{0}$, the parameters $A,\;B$ and $C$, and $g_{i}$ for each of the state equations. As shown in [25], the key length is 39 decimal digits, with a key space of 10^39^. The order of the tensor product can be used as an additional key, which has 18 combinations (which will be introduced in detail in “Key sensitivity”).

The total key space of the proposed algorithm is larger than $10^{39} \cdot 18 > > 2^{30}$, which is large enough to withstand a brutal attack.

#### Key sensitivity

The key sensitivity of measurement matrices with 3D Lorenz has been analyzed in detail to demonstrate their advantages [15, 24–26]. Here we emphasize the key sensitivity of the order of the tensor product. Although ${\mathbf{W}}_{(n)}$ is easy to be distinguished from $D_{\varPhi n}$, the ciphertexts are still difficult to decode due to both ${\mathbf{W}}_{(n)}$ and $D_{\varPhi n}$ having three models. Hence there are a totally of 18 combinations of all ${\mathbf{W}}_{(n)}$ and $D_{\varPhi n}$. Equation (16) indicates that only three combinations are correct, with 15 wrong combinations listed in the following:

$$D_{{\varPhi_{1} }} \cdot {\mathbf{W}}_{(1)} \cdot \left( {D_{{\varPhi_{3} }} \otimes D_{{\varPhi_{2} }} } \right)^{T}$$, $$D_{{\varPhi_{2} }} \cdot {\mathbf{W}}_{(1)} \cdot \left( {D_{{\varPhi_{1} }} \otimes D_{{\varPhi_{3} }} } \right)^{T}$$, $$D_{{\varPhi_{2} }} \cdot {\mathbf{W}}_{(1)} \cdot \left( {D_{{\varPhi_{3} }} \otimes D_{{\varPhi_{1} }} } \right)^{T}$$, $$D_{{\varPhi_{3} }} \cdot {\mathbf{W}}_{(1)} \cdot \left( {D_{{\varPhi_{1} }} \otimes D_{{\varPhi_{2} }} } \right)^{T}$$, $$D_{{\varPhi_{3} }} \cdot {\mathbf{W}}_{(1)} \cdot \left( {D_{{\varPhi_{2} }} \otimes D_{{\varPhi_{1} }} } \right)^{T}$$, $$D_{{\varPhi_{1} }} \cdot {\mathbf{W}}_{(2)} \cdot \left( {D_{{\varPhi_{2} }} \otimes D_{{\varPhi_{3} }} } \right)^{T}$$, $$D_{{\varPhi_{1} }} \cdot {\mathbf{W}}_{(2)} \cdot \left( {D_{{\varPhi_{3} }} \otimes D_{{\varPhi_{2} }} } \right)^{T}$$, $$D_{{\varPhi_{2} }} \cdot {\mathbf{W}}_{(2)} \cdot \left( {D_{{\varPhi_{1} }} \otimes D_{{\varPhi_{3} }} } \right)^{T}$$, $$D_{{\varPhi_{3} }} \cdot {\mathbf{W}}_{(2)} \cdot \left( {D_{{\varPhi_{1} }} \otimes D_{{\varPhi_{2} }} } \right)^{T}$$, $$D_{{\varPhi_{3} }} \cdot {\mathbf{W}}_{(2)} \cdot \left( {D_{{\varPhi_{2} }} \otimes D_{{\varPhi_{1} }} } \right)^{T}$$, $$D_{{\varPhi_{1} }} \cdot {\mathbf{W}}_{(3)} \cdot \left( {D_{{\varPhi_{2} }} \otimes D_{{\varPhi_{3} }} } \right)^{T}$$, $$D_{{\varPhi_{1} }} \cdot {\mathbf{W}}_{(3)} \cdot \left( {D_{{\varPhi_{3} }} \otimes D_{{\varPhi_{2} }} } \right)^{T}$$, $$D_{{\varPhi_{2} }} \cdot {\mathbf{W}}_{(3)} \cdot \left( {D_{{\varPhi_{1} }} \otimes D_{{\varPhi_{3} }} } \right)^{T}$$, $$D_{{\varPhi_{2} }} \cdot {\mathbf{W}}_{(3)} \cdot \left( {D_{{\varPhi_{3} }} \otimes D_{{\varPhi_{1} }} } \right)^{T}$$, $$D_{{\varPhi_{3} }} \cdot {\mathbf{W}}_{(3)} \cdot \left( {D_{{\varPhi_{2} }} \otimes D_{{\varPhi_{1} }} } \right)^{T}$$.

The average MSE of the 15 wrong combinations is 7.2895 × 10^3^. It is clear from the MSE values of the different combinations that the proposed system is sensitive to the order of the tensor product.

**Fig. 11:**
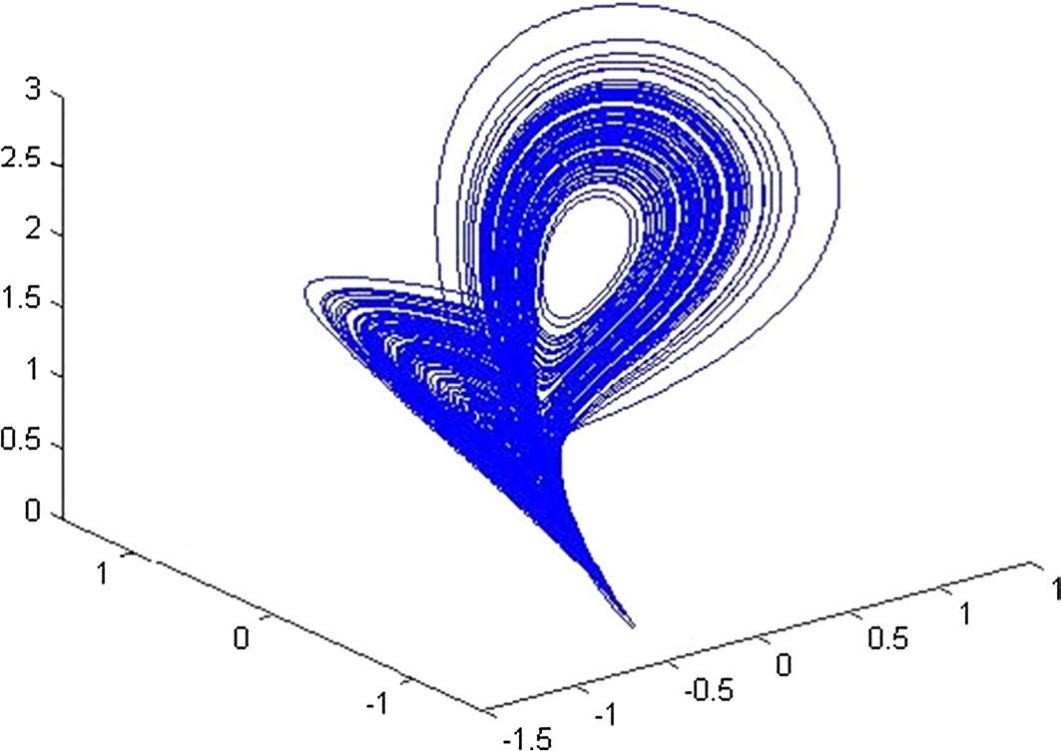
3D distribution of Lorenz system

The 3D Lorenz system is similar to that in [26]. The 3D image is encrypted with initial conditions $U_{0} = 0.1,\;V_{0} = 0,\;W_{0} = 0,\;$ and parameters *A* = 10, *B* = 28, *C* = 8/3, and *g*1 = *g*2 = *g*3 = 0.01. The maximum Lyapunov value according to the parameters and initial conditions is 0.8024 (larger than 0), the system is chaotic. The 3D distribution of the Lorenz system is also depicted in Fig. 11. Table 5 gives the sensitivity of each parameter. Any change in a parameter greater than its sensitivity will prevent an eavesdropper from decrypting message.

**Table 5:** Key sensitivity of measurement matrices with 3D Lorenz

Keys	$\Delta U_{0} = 10^{ - 6}$	$\;\Delta V_{0} = 10^{ - 6}$	$\;\Delta W_{0} = 10^{ - 6}$	$\;\Delta A = 10^{ - 4}$	$\;\Delta B = 10^{ - 4}$	$\;\Delta C = 10^{ - 4}$	$\;\Delta g1 = 10^{ - 7}$	$\;\Delta g2 = 10^{ - 7}$	$\;\Delta g3 = 10^{ - 7}$
MSE	8.50 × 10^3^	8.39 × 10^3^	8.61 × 10^3^	9.01 × 10^3^	8.67 × 10^3^	8.22 × 10^3^	8.96 × 10^3^	9.00 × 10^3^	8.65 × 10^3^

### Resistance against attacks

Common attacks include known-plaintext attack and chosen-plaintext attacks. It has been proven [10, 11] that despite the linearity of its encoding, CS may be used to provide a limited form of data protection. The TCS used in the paper is a multi-linear (non-linear) extension of the traditional CS, which further enhances the anti-attack ability. Although the attackers have access to some plaintext-ciphertext pair, they will be unable to reproduce the system without knowledge of the order of tensor product. Furthermore, the 3D Lorenz, which was introduced in the system, has the ability to withstand these common attacks [25, 26]. Therefore, the proposed system based on TCS with 3D Lorenz has the ability to resist these common attacks.

**Fig. 12:**
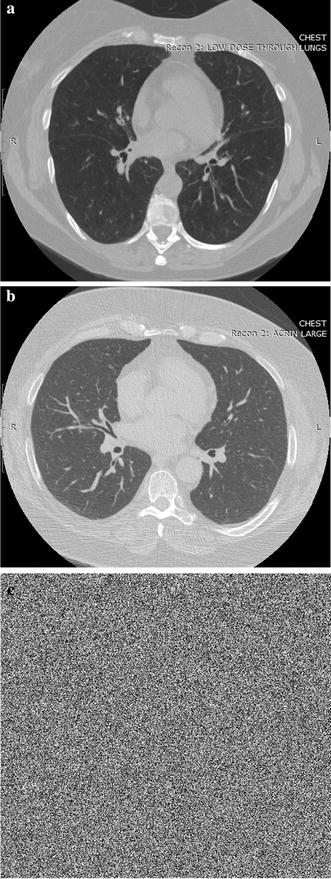
Decryption result of known-plaintext attack with the keys generated from a chosen CT sequence.** a** Original image.** b** Image used for attack.** c** Decrypted image with fake decryption keys

We ran an experiment on known-plaintext attack to evaluate the anti-attack performance. The decryption result of known-plaintext attack with the keys generated from a chosen CT sequence, which was treated as fake tensor, is displayed in Fig. 12. The frame 256 of the original and fake CT sequences are shown in Fig. 12a, b, respectively. The attack result using fake decryption keys with all correct parameters is shown in Fig. 12c. It can be seen that the retrieved image is noise-like signal. We first analyze the number of pixels rate: all pixels assumed to be an even distribution, because the retrieved image was noise-like signal. The unified average changing intensity was also used to evaluate the difference among the original and decrypted images:

25 $$\xi = \frac{1}{{M_{1} M_{2} M_{3} }}\sum\limits_{k = 1}^{{M_{3} }} {\sum\limits_{j = 1}^{{M_{2} }} {\sum\limits_{i = 1}^{{M_{1} }} {\left| {{\mathbf{\mathcal{A}^{\prime}}}(i,j,k) - {\mathbf{\mathcal{A}}}(i,j,k)} \right|} } }$$

We used the gray level to represent intensity, ranging from 0 to 255. Because the intensity value per pixel of the tensor was up to $\xi = 80$, we assume that there is a substantial difference between the original and retrieved images.

## Conclusions

In this paper, we proposed an algorithm of simultaneous encryption and compression, as applied to 3D CT volumes. This scheme has the advantages of TCS and 3D Lorenz. Its outstanding advantage is that it achieves a high precision of decryption at a big compression ratio. The security of the proposed algorithm conformed to the requirements of the common encryption technology. Moreover, the unfolding mode, which is a unique feature of the tensor product, can be used as an additional secrete key other than traditional encryption algorithms to make unauthorized decryption harder.
